# Misalignment of Career and Educational Aspirations in Middle School: Differences across Race, Ethnicity, and Socioeconomic Status

**DOI:** 10.3390/socsci5030035

**Published:** 2016-07-28

**Authors:** Brea L. Perry, Elizabeth Martinez, Edward Morris, Tanja C. Link, Carl Leukefeld

**Affiliations:** 1 Department of Sociology, Indiana University, Ballantine Hall 744, 1020 E. Kirkwood Ave., Bloomington, IN 47405-7103, USA; 2 Department of Sociology, University of Kentucky, 1569 Patterson Office Tower, Lexington, KY 40506, USA; 3 Department of Sociology & Criminal Justice, Kennesaw State University, 402 Bartow Ave, Kennesaw, GA 30144, USA; 4 Department of Behavioral Science, University of Kentucky, 111 Medical Behavioral Science Building, Lexington, KY 40506, USA

**Keywords:** disparities, career goals, educational attainment, socioeconomic status, Latino, race and ethnicity

## Abstract

Misalignment of educational and career goals (i.e., educational aspirations expressed are inadequate for attaining one’s desired occupation) is associated with lower educational attainment and a lack of college readiness, and may contribute to persistent educational and employment disparities. Drawing on data from 249 sixth graders in low-income schools, this research examines misalignment between educational and career aspirations across racial and ethnic and socioeconomic groups. Findings indicate that students in low-income schools aspire to middle and upper middle class careers, but sometimes lack an understanding of the educational degrees required to achieve their goals. Latinos are significantly more likely than other groups to report misaligned aspirations, as are students in the free and reduced lunch program and those without a college-educated parent. Consequently, early gaps in misaligned career and educational goals for disadvantaged students may set them on a trajectory that perpetuates educational and occupational inequalities in this population. We discuss the programmatic implications of these findings in light of the elevated college and career planning needs of students traditionally underrepresented in higher education.

## 1. Introduction

The US labor market has changed dramatically over the past fifty years such that obtaining a college degree is now virtually a prerequisite for a middle-class career and lifestyle. As the earnings gap between those with and without a college degree have increased over time, access to higher education has become a major stratifying force in American society [1]. While educational and occupational disparities persist across race/ethnicity and socioeconomic status (SES), some groups have made progress in reducing gaps while others lag behind. Most notably, the percentage of Blacks obtaining a college degree has increased significantly since the early 1980s, while the educational attainment of Latinos has remained nearly stagnant [2,3].

Given the role of racial and ethnic gaps in higher education in determining occupational outcomes and life chances, it is critical to identify potential barriers faced by racial and ethnic minority youth and those from disadvantaged backgrounds. We propose that one mechanism of low college graduation rates among Latinos and first generation college students, in particular, is a misunderstanding and lack of planning for the specific educational requirements of middle class careers. In the current study, we examine racial and ethnic as well as socioeconomic differences in misalignment between middle school students’ educational goals and career aspirations.

## 2. Background

Misalignment of career and educational goals occurs when the minimum education required for a person’s desired occupation exceeds their educational expectations [4]. Although both educational and career aspirations have been increasing in recent generations, there remains a major “decoupling of educational and occupational plans” ([5], p. 461). Research indicates that only 43% of youth have aligned educational and career ambitions, on average, with 16% having lower educational aspirations than are required for the desired occupation [4].

Misalignment can have important implications for young adults’ educational and occupational outcomes. Research suggests that having accurate information about the educational requirements for career ambitions and plans for reaching career goals significantly influence educational attainment as well as labor market outcomes [4,6,7]. For example, among high school students, having accurate knowledge about educational requirements for career aspirations increases educational attainment [8]. In addition, students with aligned educational and occupational aspirations are more successful at creating and fulfilling plans to achieve their occupational goals [4]. In contrast, students with misaligned knowledge or unclear occupational ambitions are less likely to enroll in a four-year college [8] and have lower educational attainment overall [9,10]. Students with misaligned educational and career goals have also been demonstrated to lack information about the steps necessary to meet career aspirations [4], and consequently have worse labor market outcomes [6], including lower adult income and higher rates of unemployment [9].

Given the adverse consequences of misalignment for life chances, it is possible that gaps between educational and career goals may contribute to the persistence of social inequality. Racial and ethnic minorities and individuals from low-socioeconomic status family backgrounds have long experienced lower educational attainment and subsequently worse labor market outcomes than Whites and middle-class families [11,12]. Educational and occupational gaps are linked to key family background characteristics, such as family income, parents’ education, and parents’ occupations [13].

These differences in social capital, as posited in Bourdieu’s theory of social reproduction, leave “most members of the lower classes with little hope of achieving social mobility” ([14], p. 45) in an educational system that values and rewards the culture of the dominant class [15]. Sullivan adds, “for Bourdieu, educational credentials help to reproduce and legitimate social inequalities, as higher-class individuals are seen to deserve their place in the social structure. […] But despite the fact that lower-class pupils are seriously disadvantaged in the competition for educational credentials, the results of this competition are seen as meritocratic and therefore as legitimate. In addition, Bourdieu claims that social inequalities are legitimated by the educational credentials held by those in dominant positions. This means that the education system has a key role in maintaining the status quo.” ([16], pp. 144–45).

Disadvantaged children often have less exposure to higher education and to occupations requiring college degrees than peers whose parents attended college or whose schools place a greater emphasis on college preparation [4,17]. Consequently, knowledge (or lack of knowledge) about the importance of higher education for attaining middle class career goals, as well as an understanding of the steps required to obtain a college degree, may reproduce social class differences across generations.

Few studies have examined differences in alignment of educational and career goals across race or ethnicity and SES [8], despite the fact that misalignment may pose significant barriers to upward mobility for those in disadvantaged groups. Because racial and ethnic minority youth are more likely to grow up in households without a college-educated parent [3], and are less likely to have college-educated adult role models [18], they may disproportionately lack access to the cultural capital required to develop and implement plans for achieving higher-status occupational goals [19]. This may be true even among those who desire middle or upper class careers, as discussed by MacLeod [20]. In his seminal research, one of the studied groups of boys could not, despite endorsing the American ideology of meritocracy and optimism about their potential for upward mobility, overcome racism and class structure to achieve their hoped-for careers.

Latino youth may be particularly susceptible to misalignment between career and educational goals due to the intergenerational social and economic consequences of immigration and acculturation. For example, only 10% of Latinos currently have a college degree, compared to 25% of Whites and 33% of Asians [2]. Moreover, about 40% of Latino youth are born to parents who did not finish high school, compared to only 4% of White children. Thus, the lower rates of formal schooling among Latino parents, many of whom are first generation immigrants, leaves them with comparatively less cultural capital to pass on to their children [21]—a resource which has been shown to affect college degree attainment [22]. In addition, Latino students are more segregated than other youth of color [23], limiting access to information about higher education that is more prevalent among Whites, Asians, and other minority groups. This isolation from middle-class America provides limited exposure to the goals and values of college-bound youth, and fosters a narrow sense of educational outcomes that are possible [18].

Despite gaps in pathways to college degree attainment, there is little evidence that Latino youth have lower career aspirations relative to Whites and other minority groups. Research on Latino youth suggests that their “hoped-for” occupational aspirations reflect middle- and upper-class careers [24]. Another study that analyzed essays written by sixth-grade Mexican-American students found that the youth aspired to be doctors, lawyers, and teachers, as well as police officers and mechanics [25]. Importantly, most of these children’s parents were agricultural workers, suggesting that while a disadvantaged socioeconomic background hinders educational attainment, it may not affect children’s dreams of a middle-class life. However, it is important to compare educational and career aspirations of young people across racial and ethnic and socioeconomic groups to understand whether Latinos’ set of structural barriers is unique.

We identified only one study explicitly comparing the degree of misalignment of career and educational aspirations of youth from different racial and ethnic and socioeconomic backgrounds [4]. This study of high school students identified no significant disparities, but recommended additional research in different contexts. Most notably, early knowledge gaps that begin prior to adolescence could shape educational trajectories and career aspirations in high school. Research suggests that educational disparities across race/ethnicity and SES have been shown to emerge as early as grade school and have lasting effects [26]. Knowledge gaps that develop in childhood could affect educational decision-making in adolescence, constituting a potential mechanism of racial and SES disparities in educational and occupational outcomes into adulthood. Consequently, the U.S. Department of Education and other educational advocacy organizations recommend that college planning and career exploration begin as early as sixth grade [27]. The current research builds upon the existing study of high school students by examining children earlier in their educational trajectories.

In addition, our study augments existing work by focusing on youth in schools with high proportions of students in poverty (i.e., Title I schools, so named for the statute providing additional federal funding to schools with greater than 50% of students from low-income families). These schools are less likely to provide services and programs that increase educational aspirations and college readiness among low-income and minority youth, including a rigorous academic curriculum, career and college planning programs, and sustained academic and social supports for students [28,29]. Moreover, research suggests that low-income and racial and ethnic minority youth are disproportionately likely to rely on counselors and other school personnel for information about college and careers [30,31], and these are precisely the types of resources that are likely to be inadequate or unavailable in financially-constrained school environments [28]. Consequently, racial, ethnic, and socioeconomic disparities in misalignment may be especially pronounced in the context of low-income schools.

The current study examines educational and career aspirations among 249 sixth grade students in low-income (Title I) schools. This analysis focuses on two research questions: first, what are the career and educational goals of youth at risk for low educational attainment, and to what degree are these goals misaligned? Second, are there significant racial and ethnic or socioeconomic disparities in misalignment of career and educational aspirations? This research can identify a potentially important mechanism of the achievement gap, and help inform school or community interventions for youth and families to reduce goal misalignment among disadvantaged groups.

## 3. Data and Methods

### 3.1. Data

Analyses for this research utilize baseline data for TRY-IT! participants and controls (*n* = 249). TRY-IT! is an intervention study to help students improve their understanding of biomedical science through intensive, two-week summer camp programming and mentoring. Detailed descriptions of the intervention and study procedures are provided elsewhere [32]. Recruitment targeted schools serving large numbers of children from low-income and minority backgrounds in Lexington, Kentucky. All sixth graders from four Title I schools were invited to participate. Data were collected from consenting students who applied for the program beginning in 2007. Because students (or parents) self-selected into this science education program, the sample is probably not representative of all students in low-income schools. That is, students with higher educational aspirations, those with parents more invested in education, and those who are higher-performing academically are probably overrepresented in the sample. Consequently, misalignment between career and educational goals is probably underestimated in these results.

The current analysis uses data on every applicant (prior to any intervention) who completed the career aspirations measures (*n* = 249; 87% of the sample). Descriptive statistics reflect the purposive sampling of students from Title I schools, creating overrepresentations of racial and ethnic minorities and youth from socioeconomically disadvantaged backgrounds: 40% of participants are White, 35% are Black, 9% are Latino, 10% are Asian, and 7% are some other race; 56% qualify for a free or reduced lunch; 23% would be first-generation college students. Finally, girls are also overrepresented (59%).

### 3.2. Measures

Educational aspirations are indexed by a single item: “I want to complete at least …” with response categories being high school, trade school, two-year/community college, and four-year college. Career aspirations are measured using: “In the space below, please describe your thoughts about your future career and/or a career that appeals to you (in any field).” Each career was coded into one of six categories: health and medicine; creative and performing arts; law and criminal justice; science and technology; teaching; or other. These are not mutually exclusive since students could name up to three occupations. In addition, each career was coded for educational requirements using Bureau of Labor Statistics (BLS) guidelines [33]. These codes were then collapsed into high school, associate’s degree, Bachelor’s degree, and advanced degree, consistent with BLS definitions [34]. Master’s and doctoral degrees were combined into one category because few of the careers provided required only a Master’s degree. Misalignment is coded as a mismatch between college aspiring and college requirements for the stated career goal. In other words, misalignment is coded “yes” if a student aspires to a college degree, but it is not required, or if a college degree is required, but the student aspires to a lower level of education.

### 3.3. Analysis

Descriptive statistics are used to examine the frequency of misaligned career and educational aspirations among middle school students. Bivariate statistics are used to identify relationships between misalignment and status group characteristics, including socioeconomic status and race or ethnicity. As all variables are categorical, cross-tabulation is performed and chi-square statistics are used to test statistical significance. Because this is only the second study to directly examine status group differences in misalignment of educational and career goals, and is the first to focus on younger children and students in low-income schools, we consider this analysis exploratory. Consequently, we focus here on establishing the direction, strength, and significance of associations rather than explaining these associations or testing theory. Thus, simple bivariate statistics are appropriate. Moreover, given that these data were initially collected for another purpose (i.e., evaluation of an intervention) and rely on a self-selected sample, these analyses and findings should be taken as preliminary.

## 4. Results

As shown in Table 1, a substantial percentage of the at-risk sixth grade students in the sample aspire to high-status and well-paying careers in health and medicine (33%; e.g., doctor, nurse, psychologist) and science and technology (28%; e.g., engineer, computer programmer, biologist). In addition, roughly one-fifth of students report aspiring to careers in law or criminal justice and creative or performing arts. An additional 8% would like to be teachers. These career aspirations typically require more than a high school diploma. About 81% of careers mentioned require at least a Bachelor’s degree, and 51% require an advanced degree.

**Figure 1:**
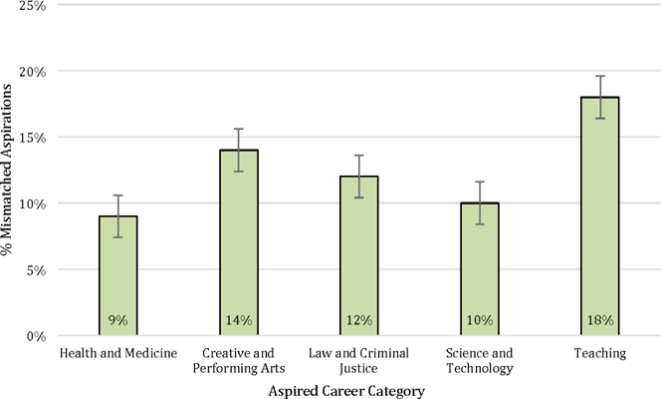
Misalignment of educational aspirations and educational requirements for career aspirations by career category (TRY-IT, *n* =249).

Our findings suggest modest misalignment of career and educational aspirations, on average. Over 10% of respondents report educational goals that fall short of the requirements for their chosen career. However, the percentage of students with misaligned goals varies across types of careers. Figure 1 presents the percent of students with mismatched goals by career field. The largest difference can be observed in teaching careers, where 18% of students who aspire to be teachers do not report a goal of finishing at least a Bachelor’s degree. However, there is less misalignment among students with career goals in health and medicine and science and technology. These differences are significant at *p* < 0.05.

**Figure 2:**
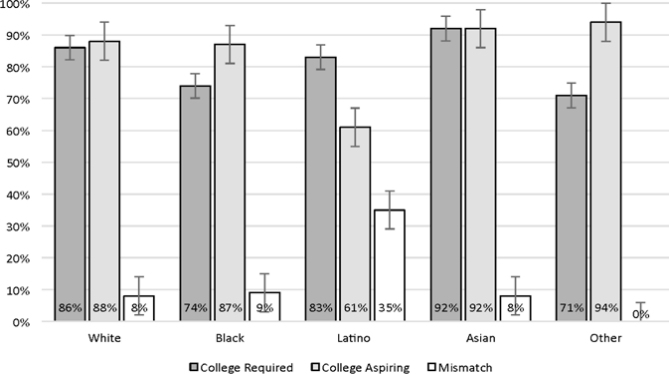
Misalignment between educational aspirations and educational requirements for career aspirations by race and ethnicity, (TRY-IT, *n* = 249).

Finally, there is evidence suggesting that misalignment of goals is unevenly distributed across social status groups, as shown in Table 2. About 35% of Latino students in the TRY-IT! sample report misaligned career and educational aspirations, compared to only 8% of Whites and 9% of Blacks (*X*^2^ = 17.38, *p* < 0.01). Interestingly, Black students’ educational goals significantly (*p* < 0.05) exceed their career aspirations, and the same pattern is observable among non-White students who are not Black, Latino, or Asian (i.e., other races). In contrast, the educational and career aspirations of Whites and Asians are almost perfectly matched. In addition, students qualifying for free or reduced lunch (*X*^2^ = 7.33, *p* <0.01) and those who would be first generation college students (*X*^2^ = 9.31, *p* < 0.01) were also disproportionately likely to have misaligned goals. As shown in Figure 2, the disparity in misaligned in misaligned aspirations among Latino youth is due almost entirely to their lower educational aspirations compared to all other racial and ethnic groups (*X*^2^ = 13.98, *p* < 0.01). That is, Latino students report career aspirations that are similar to youth of other racial and ethnic groups, but appear to lack an understanding of the educational requirements for those careers.

## 5. Discussion

These findings suggest that students in low-income schools have high career aspirations, desiring upward mobility into middle class occupations. However, they sometimes lack an understanding of the educational degrees required to achieve them. Though preliminary, our results are suggestive of variation in the extent of misalignment in career and educational goals across types of occupations. Specifically, we observe the highest rates of misalignment among students aspiring to careers in teaching, and the lowest among those desiring to be in careers in health and medicine or science and technology. This may indicate that students aspiring to the highest status careers—many of which require advanced professional degrees—are more thoughtful about what is required to attain their occupational goals, while others have less serious ideas about their future. If true, early occupational aspirations may be an indicator of college and career support services.

Most importantly, our findings suggest that while lower-SES students, would-be first generation college students, and Latino students have career aspirations that equal or exceed other students, their educational goals are disproportionately misaligned. Thus, lack of information or access to career and college planning programs, rather than limited motivation, may be a mechanism of racial/ethnic and socioeconomic disparities in college preparedness and educational attainment. This finding is critical because an early gap in knowledge of educational requirements associated with higher status career goals could play a role in academic decision-making (e.g., choosing college prep courses), affecting college readiness. If true, such patterns might be a mechanism in the American education system’s reproduction of racial or ethnic and social class inequalities. Consistent with previous research on racial, ethnic, and social class disparities in education, these patterns suggest a strong need to provide targeted career exploration and college readiness programs in low-income schools that disproportionately serve disadvantaged youth [27,28].

The degree of misalignment is particularly pronounced among Latino youth, who have significantly lower educational aspirations than students in other racial and ethnic groups. Over one-third of Latino youth express career goals that are unrealistic in light of their reported educational aspirations. This may be attributable to lower rates of college degrees or even high school diplomas among Latino parents, particularly those that are first-generation or second-generation immigrants [2]. College-educated parents transmit valuable information to their children about the importance of higher education, the links between educational attainment and middle-class careers, and the steps that are necessary to be admitted and to succeed in college [20]. Additionally, because Latino youth tend to be highly segregated into particular neighborhoods in Lexington, where this sample was drawn, they may lack access to cultural capital common in middle class families through their communities and social networks.

Additional research with nationally representative samples is the next step in understanding this pattern. Because students in TRY-IT! self-selected or were selected by parents into a science-intensive summer camp program, they may have been more informed and motivated than others. Thus, our findings may represent a conservative estimate of the problem of misaligned career and educational aspirations among sixth graders at risk for low educational attainment. Along the same lines, because these data were derived from a small convenience sample, estimates and standard errors should be interpreted with caution. In addition, because we are unable to directly test potential mechanisms of the link between race, ethnicity, SES, and misalignment, future research should empirically examine the role of cultural capital, parental and child knowledge pertaining to college and careers, and other related factors. Finally, further research is needed to understand the resources and attitudes that mitigate the misalignment problem, and to examine the long-term consequences of mismatched educational and career goals.

## 6. Conclusions

In summary, our research suggests that although the level of misalignment of career and educational aspirations in middle school is fairly modest overall, there are important status group differences. Namely, there are significantly higher rates of misalignment among three disadvantaged groups that have historically had lower rates of educational attainment—Latinos, low-income students, and youth without a college-educated parent. Our findings indicate that it is critical for schools or community organizations to provide information to Latino students and those from low-SES family backgrounds about educational requirements of high-status careers, and to assist them with developing and implementing a plan to achieve educational goals. Our findings on sixth graders suggest that this kind of intervention needs to occur much earlier than high school, when career and higher education readiness is typically addressed. Early gaps in misaligned career and educational goals for disadvantaged students may set them on a trajectory that perpetuates educational and occupational inequalities in this population.

## Figures and Tables

**Table 1:** Sample Descriptive Statistics (TRY-IT, *n* = 249).

	*n*	%
Female	146	58.6

Race/ethnicity		
White (not Latino)	99	39.8
Black	86	34.5
Latino	23	9.2
Asian	24	9.6
Other	17	6.8
Free/reduced lunch	139	55.8
Neither parent college-educated	58	23.3

Educational aspiration		
High school	9	3.6
Associate’s degree	26	10.4
Bachelor’s degree or more	214	85.9

Career aspiration (field) 1		
Health and medicine	82	32.9
Creative and performing arts	50	20.1
Law and criminal justice	44	17.7
Science and technology	70	28.1
Teaching	20	8.0

Career educational requirement		
High school	37	14.9
Associate’s degree	10	4.0
Bachelor’s degree	75	30.1
Advanced degree	127	51.0
Mismatched requirements and aspirations	26	10.4

1 Does not add up to 100% because respondents could offer more than one career choice.

**Table 2:** Educational aspirations, career educational requirements, and mismatch 1 between them by socio-demographic characteristics (TRY-IT, *n* = 249).

	College Aspiring	College Required	Mismatch	*X*^2^

	*n* (%)	*n* (%)	*n* (%)	
Gender				0.10
Female	128 (87.7)	122 (83.6)	16 (11.0)	
Male	86 (83.5)	80 (77.7)	10 (9.7)	

Race/ethnicity				17.38**
White (not Latino)	87 (87.9)	85 (85.9)	8 (8.1)	
Black	75 (87.2)	64 (74.4)	8 (9.3)	
Latino	14 (60.9)	19 (82.6)	8 (34.8)	
Asian	22 (91.7)	22 (91.7)	2 (8.3)	
Other	16 (94.1)	12 (70.6)	0 (0.0)	

Socioeconomic status				7.33**
Free/reduced lunch	111 (79.9)	108 (77.7)	21 (15.1)	
Paid lunch	103 (93.6)	94 (85.5)	5 (4.6)	

Parents’ education				9.31**
No parent college educated	44 (75.9)	48 (82.8)	12 (20.7)	
One or more college educated	169 (89.4)	152 (80.4)	13 (6.9)	

1 *X*^2^ statistic tests differences in mismatch across socio-demographic groups;

** = *p* < 0.01.
